# Ctp1 and the MRN-Complex Are Required for Endonucleolytic Rec12 Removal with Release of a Single Class of Oligonucleotides in Fission Yeast

**DOI:** 10.1371/journal.pgen.1000722

**Published:** 2009-11-13

**Authors:** Maja Rothenberg, Jürg Kohli, Katja Ludin

**Affiliations:** Institute of Cell Biology, University of Bern, Switzerland; Institut Jean-Pierre Bourgin, INRA de Versailles, France

## Abstract

DNA double-strand breaks (DSBs) are formed during meiosis by the action of the topoisomerase-like Spo11/Rec12 protein, which remains covalently bound to the 5′ ends of the broken DNA. Spo11/Rec12 removal is required for resection and initiation of strand invasion for DSB repair. It was previously shown that budding yeast Spo11, the homolog of fission yeast Rec12, is removed from DNA by endonucleolytic cleavage. The release of two Spo11 bound oligonucleotide classes, heterogeneous in length, led to the conjecture of asymmetric cleavage. In fission yeast, we found only one class of oligonucleotides bound to Rec12 ranging in length from 17 to 27 nucleotides. Ctp1, Rad50, and the nuclease activity of Rad32, the fission yeast homolog of Mre11, are required for endonucleolytic Rec12 removal. Further, we detected no Rec12 removal in a *rad50S* mutant. However, strains with additional loss of components localizing to the linear elements, Hop1 or Mek1, showed some Rec12 removal, a restoration depending on Ctp1 and Rad32 nuclease activity. But, deletion of *hop1* or *mek1* did not suppress the phenotypes of *ctp1Δ* and the nuclease dead mutant (*rad32-D65N*). We discuss what consequences for subsequent repair a single class of Rec12-oligonucleotides may have during meiotic recombination in fission yeast in comparison to two classes of Spo11-oligonucleotides in budding yeast. Furthermore, we hypothesize on the participation of Hop1 and Mek1 in Rec12 removal.

## Author Summary

A diploid zygote arises by fusion of two haploid gametes. The specific cell division leading to haploid gametes with haploid chromosome number, accompanied by recombination of genetic material, is called meiosis. It is essential for sexually reproducing eukaryotes. During meiotic prophase, shortly after DNA replication, programmed DNA double-strand breaks mark the initiation of recombination. In budding yeast, the protein responsible for DNA double-strand break formation, Spo11, creates a covalent DNA-Spo11 intermediate, which needs to be removed for subsequent recombination. Presumably, asymmetric endonucleolytic cleavage of DNA next to bound Spo11 leads to distinct DNA ends in budding yeast and mouse. Here we show that the fission yeast Spo11 homolog Rec12 is removed by endonucleolytic cleavage as well. However, only a single oligonucleotides class can be detected suggesting symmetric cleavage. We show that Rec12 removal depends on Ctp1 and the MRN-complex. Furthermore, we applied this new method to monitor DNA double-strand break repair in mutants. Until now, pulsed-field gel electrophoresis was used to monitor global DNA double-strand break formation and repair. The assay presented here provides a new tool to monitor global DNA double-strand break processing only.

## Introduction

Meiosis is a special type of cell division generating haploid gametes. One round of DNA replication is followed by two successive divisions separating homologous chromosomes (Meiosis I) and sister chromatids (Meiosis II).

Recombination during meiotic prophase ensures the formation of physical connections, called chiasmata, between the homologous chromosomes, indispensable for proper homolog separation in most species.

In prophase, before the first meiotic division, homologous chromosomes become aligned and recombination is initiated by the topoisomerase-like protein Spo11 in *Saccharomyces cerevisiae*, which catalyzes double strand break (DSB) formation by a transesterification reaction [1]. Afterwards Spo11 remains covalently attached to each 5′ end of the meiotic DSB [2],[3]. An amino acid change of tyrosine-135 to phenylalanine abolishes covalent linkage of Spo11 with DNA [1]. This covalent linkage is evolutionarily conserved in the *Schizosaccharomyces pombe* Spo11 homolog Rec12 (tyrosine-98, [1],[4]).

True topoisomerase activity (religation of DNA) has not been demonstrated for Spo11. Instead, Spo11 was shown to be excised from the DNA by endonucleolytic cleavage, resulting in free 5′ ends accessible for further strand resection [5]. The released Spo11-oligonucleotide consisted of two classes of oligonucleotides heterogeneous in length. As the most straightforward explanation for these two classes of oligonucleotides, Neale et al. hypothesized asymmetric cleavage. The remaining distinct DNA ends would present different loading platforms for recombination proteins, e.g. Rad51 and Dmc1 [5].

DSB formation does not exclusively depend on Spo11/Rec12 action, but requires multiple auxiliary proteins in both yeasts (reviewed in [6]). The evolutionarily conserved Mre11/Rad50/Nbs1 (MRN) complex, in *S. cerevisiae* termed MRX (Mre11/Rad50/Xrs1), has a central role in mitotic and meiotic DNA repair (reviewed in [7]). Any null mutant of the MRX complex in *S. cerevisiae* abolishes meiotic DSB formation [8]–[10]. Point mutations in *MRE11* or *RAD50*, e.g. *mre11S* or *rad50S*, accumulate unrepaired DSBs with Spo11 still covalently bound to the DNA [2]. Therefore the MRX complex is required not only for DSB formation, but also for removing Spo11 from meiotic DSB ends. In addition Sae2/Com1 was shown to be required for DSB repair, but not for DSB formation [11],[12]. The *sae2Δ* mutant shows a phenotype similar to *rad50S*. *In-vitro* experiments suggested a cooperative action of Sae2 and the MRX complex [13]. In *S. pombe* the MRN complex is not required for DSB formation [14]. A point mutation, also called *rad50S* in *S. pombe*, accumulates DSBs, resulting in low spore viability, which can be partially rescued by deletion of *rec12* [15],[16]. Similar to *sae2Δ* in *S. cerevisiae*, deletion of the *S. pombe* homolog *ctp1* does not affect DSB formation [17], but abolishes Rec12 removal from DNA, like the nuclease dead mutant of Rad32 (Rad32-D65N) [16].

Besides proteins directly involved in DSB formation, other meiosis-specific proteins affect DSB formation in *S. cerevisiae*, among them Red1, Mek1, and Hop1, which associate with lateral elements of the synaptonemal complex (SC) [18],[19]. The SC is formed in *S. cerevisiae* and many other eukaryotes to mediate homologous chromosome pairing. In *S. pombe* proteinaceous structures, called linear elements (LEs), are formed instead [20],[21].

*S. cerevisiae MEK1* and *HOP1* genetically interact with *RED1* [22]. Meiotic intergenic (7 to 60 fold) and intragenic (4 to 25 fold) recombination are reduced in *mek1* and *hop1* mutants, depending on the interval [23],[24]. Loss of both proteins lead to DSB reduction of 5 to 15% compared to wild type [25]–[27]. Unequal recombination between sister chromatids is increased in *mek1* and *red1* mutants, suggesting a participation of these three proteins in a “barrier to sister chromatid repair” during meiotic recombination [28].

*S. pombe* Hop1 and Mek1 localize to Rec10, a main component of the LEs and the distant homolog of *S. cerevisiae* Red1 [21],[29]. As in *S. cerevisiae*, Mek1 was identified as a meiosis-specific kinase participating in the regulation of meiotic cell cycle progression in fission yeast [29]. Intergenic recombination was reduced in a *mek1* mutant compared to wild type [29].

Here we present evidence for the removal of *S. pombe* Rec12 from DNA by endonucleolytic cleavage. Unlike in *S. cerevisiae*, *S. pombe* Rec12-oligonucleotides were found to be homogeneous in length, which may indicate symmetric cleavage. Rec12 removal depends on Ctp1, Rad50, and the nuclease activity of Rad32. Furthermore, we present evidence that Rec12 removal in a *rad50S* mutant can be partially restored by deletion of *hop1* or *mek1* with subsequent DSB repair.

## Results

### Rec12-oligonucleotides are byproducts of meiotic DSB repair, and their removal depends on Ctp1 and the MRN-complex

*pat1-114* meiosis has the advantage that cells enter and proceed through meiosis in a highly synchronous way [30]. Although *pat1-114* meiosis leads to chromosome segregation defects in the first meiotic division [31], it has been used systematically for studying the timing of early meiotic events, including DSB formation and repair [4],[15]. Strains carrying the *pat1-114* mutation were arrested in G1 by nitrogen starvation. A temperature shift to 34°C induces meiosis.

**Figure 1:**
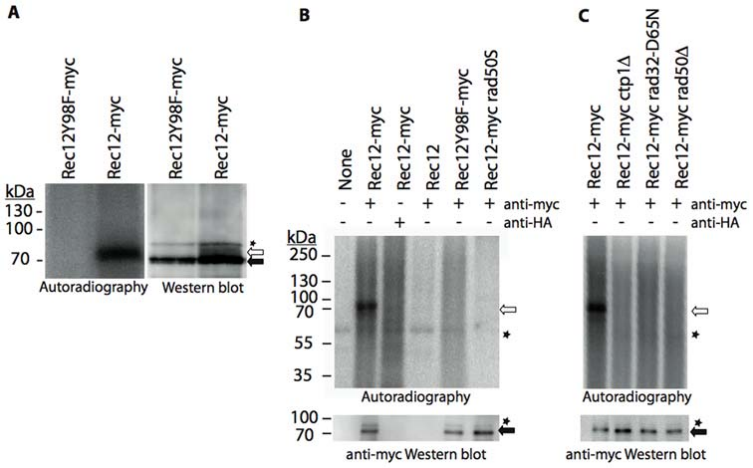
Rec12-oligonucleotides are by-products of endonucleolytic cleavage. (A) Rec12myc and Rec12Y98Fmyc immunoprecipitates from *pat1-114* meiotic time-courses at 4h after temperature shift were first analyzed by SDS-PAGE and autoradiography after TdT labeling (left panel). The same filter was subsequently subjected to Western blot analysis (right panel). (B) *pat1-114* meiotic extracts from strains *rec12myc* (KLY67), *rec12^+^* (22-859), *rec12Y98Fmyc* (KLY384), and *rec12myc rad50S* (MR399) were immunoprecipitated and TdT-labeled (upper panel). Western blot analysis revealed presence of Rec12myc (lower panel). (C) Analogously, mutants of the MRN complex and Ctp1 were investigated: *ctp1Δ* (MR475), *rad32-D65N* (MR479), and *rad50Δ* (MR481). Open arrow indicates Rec12-oligonucleotide, filled arrow indicates free Rec12myc, and asterisks correspond to non-specific signals.

Meiotic DSB formation starts at 3 hours after meiotic induction, reaches a maximum at 4 hours, and ends at 5 hours [32]. Rec12 expression was monitored in the *pat1-114* meiosis by Western blot analysis. Pre-meiotic DNA replication and completion of meiotic division was checked by FACS analysis and DAPI staining (Figure S1, data not shown). As expected, myc epitope-tagged Rec12 taken four hours after meiotic induction migrated at 68 kDa (Figure 1A, right panel). A more slowly migrating protein at 75 kDa was also detected. Presumably, the 68 kDa protein corresponded to free Rec12. We examined whether the 75 kDa protein could be a removed Rec12-oligonucleotide compound, like it was found in budding yeast [5].

Terminal transferase (TdT) catalyzes the addition of deoxynucleotides to 3′ hydroxyl termini of DNA molecules. Treating immmunoprecipitated Rec12 with TdT together with a chain termination desoxynucleotide analog (α-^32^P-cordycepin) revealed that the 75 kDa protein is a Rec12-oligonucleotide (Figure 1A, left panel). In a Rec12Y98Fmyc active site mutant no DSB formation occurs [4] and no Rec12Y98F-oligonucleotide appeared. Rec12-oligonucleotide appearance was specific for Rec12myc and anti-myc antibody (Figure 1B). Neither untagged Rec12 nor Rec12myc immunoprecipitation with an anti-HA antibody led to the detection of Rec12-oligonucleotide.

In a *rad50S* mutant, where DSBs are not repaired [15] and Rec12 stays covalently attached to the DNA, no Rec12-oligonucleotide was detectable (Figure 1B). Furthermore, Rec12-oligonucleotide was not detectable in *rad50Δ*, *ctp1Δ*, and *rad32-D65N* nuclease dead mutant (Figure 1C). This reveals that Ctp1 and the MRN complex are required for removing Rec12 from the DNA.

**Figure 2:**
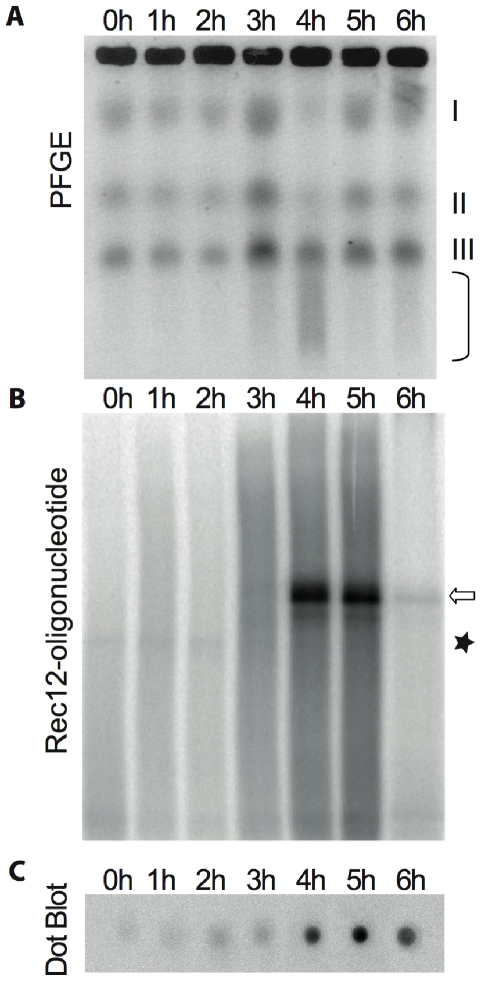
Parallel analysis of Rec12-oligonucletide and DSB formation. (A) *pat1-114* samples were analyzed for meiotic DSB formation by resolving chromosomal DNA by PFGE. Intact chromosomes (chromosome I 5.7 Mb, II 4.6 Mb, III 3.5 Mb) as well as smears resulting from breakage (parenthesis) are indicated. (B) Samples from the same time course experiment were analyzed for Rec12-oligonucleotide appearance. Open arrow indicates Rec12-oligonucleotide, asterisk corresponds to a non-specific signal. (C) Furthermore, immunodetection of Rec12myc was performed in a dot-blot assay. Lanes refer to hours after meiotic induction.

Investigation of meiotic DSB formation by pulsed-field gel electrophoresis (PFGE) and Rec12-oligonucleotide appearance in the same *pat1-114* meiotic culture revealed similar timing of these events (Figure 2). DSBs became visible at around 3 hours after meiotic induction and reached a maximum at 4 hours. During further meiotic progression, DSBs became repaired and intact chromosomes reappeared (Figure 2A). Shortly after the start of DSB formation Rec12-oligonucleotide appeared, reached a maximum at 4 to 5 hours, and disappeared at later time points (Figure 2B). Rec12myc expression was followed by immunodetection in dot-blot analysis (Figure 2C).

### Length determination of the oligonucleotide covalently bound to Rec12

**Figure 3:**
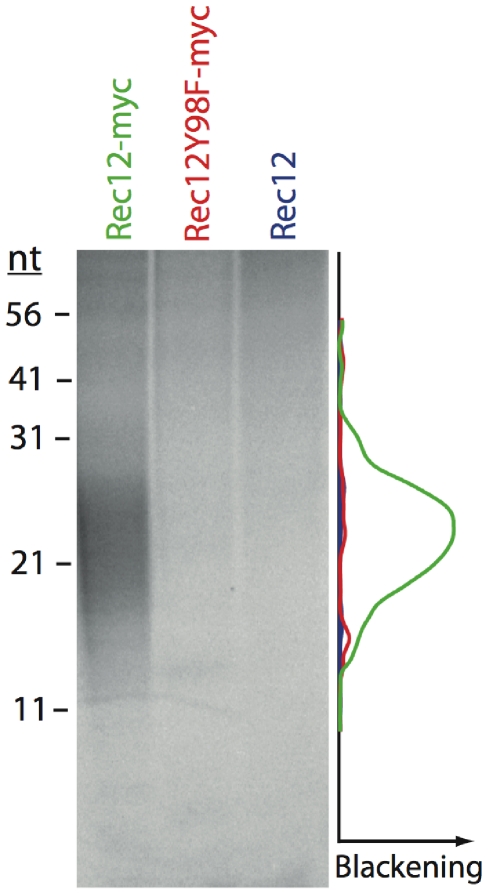
Length determination of oligonucleotides bound to Rec12. Rec12myc (green), Rec12Y98Fmyc (red), and untagged Rec12 (blue) immunoprecipitates from *pat1-114* meiotic time courses at 4h after temperature shift were TdT-labeled and deproteinized with Pronase. Separation of the labeled oligonucleotides on a 20% denaturing polyacrylamide gel revealed a length between 17 and 27 nucleotides. The radioactivity profiles (AIDA quantification software) of each lane are given in the respective color.

The mass difference of about 7 kDa between free Rec12 and Rec12-oligonucleotide seen in the Western blot analysis (Figure 1A, right panel), suggested an oligonucleotide length of approximately 23 nucleotides (mean nucleotide mass of 308 Da). To elucidate further the length of this oligonucleotide, immunoprecipitated Rec12 extract was deproteinized with pronase after TdT labeling and separated on a 20% denaturing polyacrylamid gel (Figure 3). Oligonucleotides of known length were also labeled with TdT, and used as size markers. We detected a signal between 17 to 27 nucleotides (Figure 3), which is in accordance with the results from Western blot analysis and autoradiography (Figure 1A). The signal was specific for the myc-tag and Rec12 activity (Figure 3). Thus, Rec12 is removed from the DNA by endonucleotic cleavage, which releases Rec12 bound oligonucleotides with a mean length of about 22 nucleotides.

### Analysis of Rec12-oligonucleotide in *hop1* and *mek1* mutants

**Figure 4:**
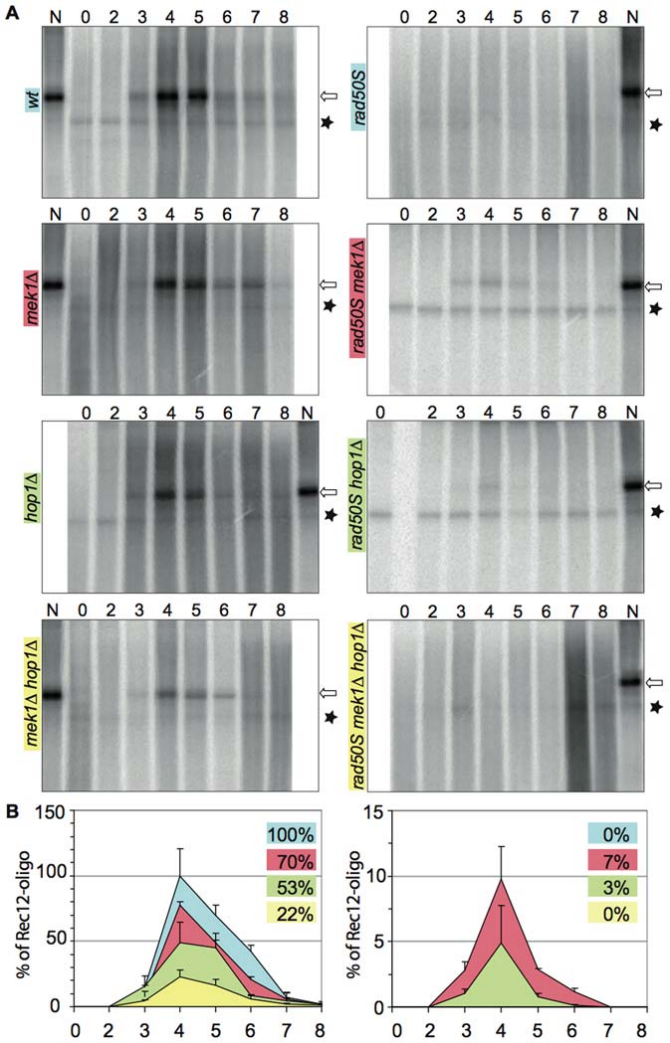
Rec12-oligonucleotides in *hop1* and *mek1* mutants. (A) Rec12-oligonucleotides (open arrow) were immunoprecipitated from extracts of cells taken at the indicated time points from a meiotically induced *pat1-114* culture. Samples were TdT labeled, separated by SDS PAGE and analyzed with a PhosphorImager. The strains were: *rec12myc* (KLY67), *rec12myc mek1Δ* (MR442), *rec12myc hop1Δ* (MR395), *rec12myc mek1Δ hop1Δ* (MR440), *rec12myc rad50S* (MR399), *rec12myc rad50S mek1Δ* (MR450), *rec12myc rad50S hop1Δ* (MR391), and *rec12myc rad50S mek1Δ hop1Δ* (MR446). N is a normalization standard (see Materials and Methods). The asterisk indicates a non-specific signal. (B) Quantification of Rec12-oligonucleotides from (A) by using the AIDA 1D quantification software. The graphs represent the normalized mean values of Rec12-oligonucleotide from at least three experiments. The error bars indicate the standard error of the mean.

Investigations of DSB formation and repair by PFGE in a *rad50S hop1Δ* mutant indicated partial DSB repair (our unpublished data). Direct analysis of Rec12 oligonucleotide appearance in time-course experiments of *rad50^+^* and *rad50S* strains in combination with *hop1Δ*, *mek1Δ*, or *hop1Δ mek1Δ*, would clarify whether this partial repair was actually occurring by Rec12-oligonucleotide removal as in *hop1^+^* (Figure 4). Radiolabeled Rec12-oligonucleotide was analyzed after separation with a PhosphorImager and quantified by the AIDA software. The amount of Rec12-oligonucleotide at each time point was normalized to a standard meiotic sample N (see Materials and Methods). The summation of Rec12-oligonuclotide values observed throughout the time-course in the *pat1-114* strain was defined as 100%. Values of Rec12-oligonucleotide in mutant strains were estimated accordingly. We used equal amounts of cells for the IP experiments (see Materials and Methods). Furthermore, the wild type and mutant strains used for the experiments in Figure 4, showed overall Rec12myc abundance with similar kinetics and amounts throughout the time-courses (dot blots, see Figure S2).

Rec12-oligonucleotide release occurred in the mutant strains with comparable timing to the *pat1-114* strain and started at 3 hours, reached a maximum at 4 to 5 hours, and diminished afterwards. Less Rec12-oligonucleotide was detected in *mek1Δ* (70%), *hop1Δ* (53%), and *hop1Δ mek1Δ* (22%) mutants (Figure 4A and 4B, left panels).

As shown above, in a *rad50S* mutant no Rec12-oligonucleotide was detectable. Remarkably, Rec12-oligonucleotides were detectable to 7% and 3% in strains carrying *mek1Δ* or *hop1Δ*, respectively (Figure 4A and 4B, right panel). No Rec12-oligonucleotide was detectable in a *rad50S hop1Δ mek1Δ* mutant. These findings indicate that in the *rad50S* mutation absence of the LE associated proteins Hop1 or Mek1 leads to at least some removal of Rec12 from the chromatide fragments.

### Analysis of spore viability and homologous recombination

In the *rad50S* mutant, where unrepaired DSBs accumulate [15], low spore viability was detected [16]. We confirmed the low spore viability at the restrictive temperature of the *rad50S* mutant (6%, Table 1). Surprisingly, the spore viability was increased in homozygous crosses of *rad50S mek1Δ* (17%) and *rad50S hop1Δ* (20%) in comparison to *rad50S*. The *rad50S mek1Δ hop1Δ* mutant showed *rad50S* single mutant level (8%). Furthermore, we studied intragenic recombination (gene conversion) in the non-hotspot interval *ade7-50*×*ade7-152* at the restrictive temperature of 34°C for *rad50S* using the same material from the crosses for spore viability determination (Table 1). Notably, conversion frequency in the homozygous *rad50S* crosses was reduced about 70-fold compared to wild type. *rad50S mek1Δ* and *rad50S hop1Δ* mutants were only 40-fold reduced compared to wild type, whereas the conversion frequency in the *rad50S mek1Δ hop1Δ* crosses was comparable to the *rad50S* value.

**Table 1:** Spore viability and intragenic recombination for wild type, *rad50S*, *hop1D*, and *mek1D* mutants.

Genotype^a^	Spore viability^b^ (mean± SEM)	fold reduction to wild type	Intragenic recombination^b^^,^ c (ppm±SEM)	fold reduction to wild type
wild type (1–26×51–2011)	89.1±2	1	560±18	1
*rad50S* (MR463×MR467)	6.3±0.7	14	7.8±1.4^d^	72
*rad50S mek1Δ* (MR465×MR469)	17.1±0.5	5.2	12.4±1.5^d^	45
*rad50S hop1Δ* (MR464×MR468)	19.9±2.5	4.5	12.7±2.4^d^	44
*rad50S mek1Δ hop1Δ* (MR466×MR470)	7.7±0.6	11.5	9.2±0.5^d^	61

a For detailed genotypes see Table S1.

b Crosses were carried out at 34°C.

c Intragenic recombination was measured in at least 3 crosses in the interval *ade7-50*×*ade7-152* and given as the mean ppm ± standard error of the mean (SEM, ppm: number of prototrophic recombinants per million viable spores).

d At least 30 *ade^+^* prototrophic colonies were counted in total per mutant.

### Restoration of Rec12 removal by *hop1Δ* and *mek1Δ* in *rad50S* depends on Ctp1 and Rad32 nuclease activity

**Figure 5:**
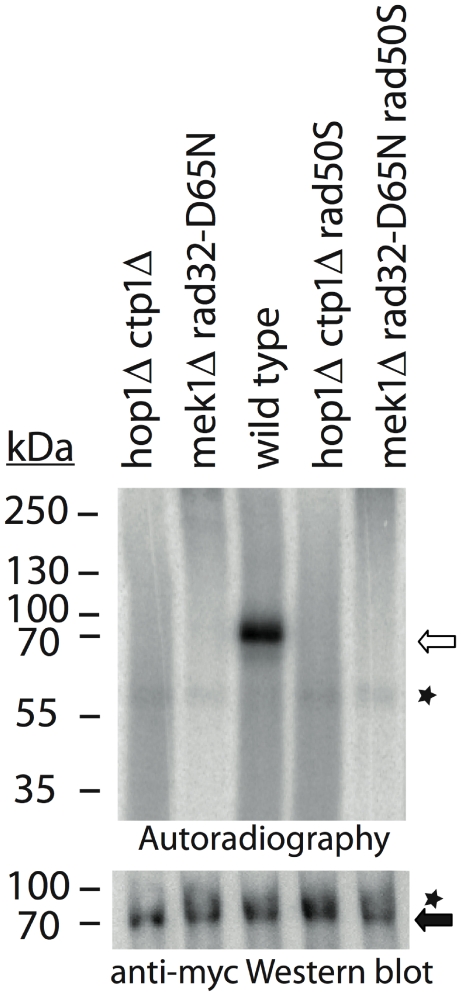
Rec12-oligonucleotide removal in *hop1Δrad50S* and *mek1Δrad50S* depends on Ctp1 and Mre11 nuclease activity. Rec12myc immunoprecipitates from *pat1-114* meiotic time-courses at 4h after temperature shift were analyzed by autoradiography after TdT labeling. Meiotic *pat1-114* extracts from the strains *hop1Δ ctp1Δ* (MR477), *mek1Δ rad32-D65N* (MR487), wild type (MR388), *hop1Δ ctp1Δ rad50S* (MR482), and *mek1Δ rad32-D65N rad50S* (MR484) were immunoprecipitated for Rec12myc and TdT-labeled (upper panel). Western blot analysis revealed presence of Rec12myc (lower panel). The open arrow indicates Rec12myc-oligonucleotide, the filled arrow Rec12myc, and asterisks correspond to non-specific signals.

To elucidate whether Ctp1 and the MRN-complex contribute to the observed Rec12 removal in *rad50S hop1Δ* and *rad50S mek1Δ*, further mutants were analysed. There was no Rec12-oligonucleotide detectable in *rad50S hop1Δ ctp1Δ*, *rad50S mek1Δ rad32-D65N*, *hop1Δ ctp1Δ*, or *mek1Δ rad32-D65N* (Figure 5). Therefore, it seems that Ctp1 and the nuclease activity of Rad32 are required for removal of Rec12 from DNA in *rad50S hop1Δ*, *rad50S mek1Δ*, as well as in *rad50^+^* (Figure 1C). Moreover, neither deletion of *hop1* nor *mek1* is able to restore Rec12 removal in *ctp1Δ* or *rad32-D65N*; the suppression is specific for *rad50S*.

## Discussion

### Endonucleolytic Rec12-oligonucleotide removal results in a single oligonucleotide class

Is was hypothesized that *S. cerevisiae* Spo11 is removed by asymmetric cleavage, resulting in two classes of oligonucleotides, heterogeneous in length (10–15 and 24–40 nucleotides, respectively). Here we show that fission yeast Rec12 is removed from DNA by endonucleolytic cleavage, releasing one class of Rec12-oligonucleotide only (Figure 1 and Figure 3). Appearance of this repair byproduct correlates with DSB presence (Figure 2). The length of covalently bound DNA to Rec12 is in the range of 17 to 27 nucleotides (Figure 3), which is larger than the short class (10 to 15 nucleotides), but not as large as the longer class (24 to 40 nucleotides) of Spo11 bound oligonucleotides in *S. cerevisiae*. The signal of TdT-labeled oligonucleotides suggested one class with an average length of 22 nucleotides in *S. pombe* (Figure 3), which is in accordance with the more slowly migrating Rec12-oligonucleotide in Western blot analysis (Figure 1A). This interpretation is strengthened by the single peak profile of the oligonucleotide signal (Figure 3). However, we cannot ascertain that the released oligonucleotides fall into two discrete length classes with mean lengths differing by only few nucleotides.

During submission of this paper an analogous study investigating the removal of Rec12 after DSB formation in *S. pombe* has been published [33]. Although the experimental setting slightly differs, one class of oligonucleotides with a mean length of 22 nucleotides was found as well.

A single class of oligonucleotides would lead to the prediction that Rec12 is, contrary to Spo11 in *S. cerevisiae* and mouse [5], removed by symmetrical cuts from the chromatid fragments. What causes and consequences may symmetric Rec12 removal have for subsequent DSB processing?

For initiation of meiotic DSB formation, several meiosis-specific proteins are required. In *S. cerevisiae*, several protein sub-complexes assemble and load in a regulated fashion at emerging DSB sites [34],[35]. In addition, the MRX complex (Mre11/Rad50/Xrs1) loads independently of Spo11 to meiotic chromatin [36] and is indispensable for DSB formation [8]–[10]. In *S. pombe*, only few of the *S. cerevisiae* proteins required for DSB formation are conserved. Instead, several *S. pombe* specific proteins have been identified. Sterical constraints based on asymmetric cleavage present in *S. cerevisiae* may thus not exist in *S. pombe*.

Neale et al. suggested the following model for the presumed asymmetric Spo11 removal in *S. cerevisiae*: the shorter Spo11-oligonucleotide would be released for immediate resection, whereas the longer one would remain basepaired leading to a delay of resection. This creates timely ordered accessibility of the 3′OH ends, facilitating an orchestrated preferential strand invasion into a chromatid of the homologous chromosome. Rad51 and Dmc1 could be loaded differentially on the distinct DNA ends, in accordance with the finding of side-by-side formation of Rad51 and Dmc1 foci [37].

Physical analysis of joint molecules (JMs) in budding yeast, revealed that the majority of JMs are formed between homologous chromosomes [38],[39]. Furthermore, Dmc1 specifically promotes interhomolog JMs [38]. In addition, the *dmc1* mutant shows a meiotic arrest with extensively resected DSB ends [40]. In fission yeast, however, sister chromatid JMs outnumber homolog JMs by a factor of four [41]. The *S. pombe dmc1Δ* mutant forms and repairs DSBs, and meiotic prophase is not arrested [14]. Weak reductions of recombination in the single mutants, but strong reduction in *rad51Δ dmc1Δ*, indicates redundant meiotic functions of Dmc1 and Rad51 [42]. A differential loading of Rad51 and Dmc1 may not occur in fission yeast, and thus an asymmetric Rec12 removal would not be necessary.

### Proteins involved in Rec12 removal

It has been suggested, that Sae2/Com1, as well as the Rad50 and Mre11 components of the MRX complex, are required for Spo11 removal in *S. cerevisiae* [5]. However, since these proteins are also required for DSB formation, investigation of their actual requirement for Spo11 removal from the DNA is difficult in this organism. On the contrary, the *S. pombe* MRN complex (Rad32/Rad50/Nbs1) is not required for DSB formation [14], but obviously Rad50, as well as Ctp1 and Rad32 endonuclease activity are essential for Rec12 removal (Figure 1C). These findings are in accordance to experiments showing Rec12 retention on chromosomal DNA in *ctp1Δ* or *rad32D65N* nuclease dead mutant [16]. Thus, we conclude that Ctp1 and Rad32 are key players in the removal of Rec12 from the DNA.

### Reduced amounts of Rec12-oligonucleotide in *hop1* and *mek1* mutants

The Rec12-oligonucleotide amounts in *hop1Δ*, *mek1Δ*, and *hop1Δ mek1Δ* strains were reduced (see Figure 3A). Probably, this reduction of repair byproduct is due to reduced DSB formation. Since DSB formation and repair are simultaneous processes, full quantification of DSB formation is not possible in these mutants.

Rec7 is required for meiosis specific DSB formation and aggregates in foci on LEs in immuno-stained nuclear spreads [43]. The amount of Rec7 foci in *hop1Δ* and *mek1Δ* mutants was reduced, suggesting less Rec7 cooperation with Rec12 for DSB formation [43]. Furthermore, Rad51 foci, marking DSB repair sites, were also reduced in *hop1Δ* and *mek1Δ* [43]. Taken together, these findings strengthen the argument for reduction of DSB formation in these mutants.

However, alternative Rec12 removal that does not involve endonucleolytic cleavage, for instance involvement of a phosphodiesterase like Tdp1, cannot be excluded. The *S. cerevisiae* homolog of Tdp1 has been shown to participate in Top2 removal, which like Spo11/Rec12 is bound to the 5′ ends of DNA [44].

### Rec12 is partially released in *rad50S hop1Δ and rad50S mek1Δ* mutants

We did not detect Rec12-oligonucleotide in a *rad50S* mutant (Figure 4). This finding is in agreement with the described accumulation of unrepaired DSBs and low spore viability [15],[16]. Surprisingly, Rec12-oligonucleotide was detectable in low amounts in *rad50S mek1Δ* and *rad50S hop1Δ*. In addition, spore viability and intragenic recombination in both mutants were increased compared to *rad50S* (Table 1). Together these findings indicate partial restoration of Rec12 removal in *rad50S* by *hop1Δ* or *mek1Δ* deletion. Furthermore, we were able to show that the observed suppression depends on Ctp1 and Rad32 nuclease activity, like Rec12 removal in *rad50^+^*. Obviously, once Rec12 is removed, DSB repair is possible resulting in elevated intragenic recombination and the increased spore viability.

No Rec12-oligonucleotide was detectable in *rad50S hop1Δ mek1Δ*. We interpret this finding differently than the one in *rad50S*. The amount of Rec12-oligonucleotide detected in the *rad50^+^ mek1Δ hop1Δ* mutant was reduced about 2-fold compared to *rad50^+^ hop1Δ* (see above). Assuming a 2-fold reduction in *rad50S mek1Δ hop1Δ* compared to *rad50S hop1Δ*, the Rec12-oligonucleotide amount would probably be below the detection limit.

In *S. cerevisiae*, genetic interactions between components associated with the SC's lateral elements (Mek1, Hop1, Red1), Sae2, and the MRX complex have been reported: Firstly, a *hop1Δ* mutant suppresses the *rad50S* spore lethality [26], and secondly, a *hop1* and *red1* deletion suppresses a *sae2Δ* sporulation defect [45]. *In-vitro* experiments validate the cooperative action between the MRX complex and Sae2 [13],[46]. Furthermore, co-localization of Sae2 and Mre11 in nuclear spreads is impaired in the *rad50S* mutant, suggesting a role of Rad50 in mediating the interaction between the MRX complex and Sae2 in meiosis [47].

Recently, *S. pombe* Ctp1 and the nuclease activity of Rad32 (homologs of *S. cerevisiae* Sae2 and Mre11) were shown to be required for Rec12 removal from the DNA, suggesting conserved action [16]. The *S. pombe rad50S* mutation is, in contrast to the analogous mutation in budding yeast, a temperature sensitive allele. At the restrictive temperature (34°C) the activation of the endonuclease needed for the removal of Rec12 is probably impaired. This might be either due to a conformational change within the M(Rad50S)N complex and/or lost interactions with associated proteins, e.g. Ctp1.

How might a deletion of Mek1 or Hop1 provoke endonuclease activity in the *rad50S* background? The association of Hop1 and Mek1 with the LEs might indicate their participation in meiosis-specific chromatin organization to promote chromosome pairing. The endonuclease activity required for removing Rec12 from the broken DNA is, as described above, provided by the cooperative action of the MRN complex and Ctp1 (Figure 1C). We suggest that the presence of Hop1 and Mek1 in a *rad50S* mutant impairs the interaction between the MRN complex and Ctp1, and thus Rec12 remains covalently bound to the DNA at the site of DSB. Absence of Hop1 or Mek1 would then loosen the chromatin organization and advance the encounter of MRN and Ctp1, causing some Rec12 removal. Obviously, the same proteins are responsible for Rec12 removal in wild type, *rad50S hop1Δ* and *rad50S mek1Δ* meiosis since no Rec12 removal was detectable in *rad50S hop1Δ ctp1Δ* or *rad50S mek1Δ rad32D65N* triple mutants (Figure 5).

In summary, the single class of Rec12-oligonucleotide described adds to the list of differences between the two model organisms *S. pombe* and *S. cerevisiae*. Perhaps, this also occurs in other eukaryotes, particularly in those that do not possess Dmc1 homologs, e.g. *Drosophila melanogaster* and *Caenorhabditis elegans* [48]. We have demonstrated genetic interaction between *hop1* and *rad50S* in *S. pombe*. In addition, we found a genetic interaction between *mek1* and *rad50S*. In both cases, the partial restoration of Rec12-oligonucleotide formation as a byproduct of DSB processing does occur. Moreover, we were able to shed light on the participation of Ctp1 and Rad32 in Rec12 removal from DNA ends after DSB formation. Rec12 removal seems to be achieved by joint action of proteins, including Ctp1, Rad50, and Rad32. Both potential endonucleases, Ctp1 and Rad32, are required for cleavage. Until now, PFGE was used to monitor global DSB formation and repair. By measuring the appearance of the Rec12-oligonucleotide byproduct as presented here, a new tool for monitoring global DSB processing is now available in *S. pombe*.

## Materials and Methods

### Culture methods and yeast strains

Media and general methods are described in [49],[50]. Synthetic medium, EMM with 2% glucose and EMM without nitrogen source (EMM-N) with 1% glucose were described in [51]. 75 and 15 mg/liter adenine were added to EMM and EMM-N.

Meiotic time-course experiments were carried out as described elsewhere [30]. Samples were checked by FACS analysis and DAPI staining for synchronicity and completion of meiosis (see Figure S1).

*S. pombe* strains are listed in Table S1. The *rec12Δ::hygR* deletion was constructed according to the Baehler method [52]. Rec12myc functionality has been tested previously [53]. The *rec12Y98Fmyc* allele was constructed as follows: an integrative plasmid (pYC36A, [31]), containing 2 kb of 5′ non-translated promoter region of *rec12*, the *rec12* ORF, and 13 copies of the myc epitope tags (from the pFA6a series [52]), was targeted for site-directed mutagenesis (QuikChange Lightning Site-directed mutagenesis Kit from Stratagene) with the primers #KL241 (5′-CGAAAATTCAGATTTAAGTTCTAATTACATTTGCAGAGATATCTATTTCAGAGATGTAGATTTATTCAAG-3′) and #KL242 (5′-CTTGAATAAATCTACATCTCTGAAATAGATATCTCTGCAAATGTAATTAGAACTTAAATCTGAATTTTCG-3′), introducing an *Eco* RV restriction-recognition site together with the amino acid change tyrosine to phenylalanine at position 98. The plasmid was stably integrated at the *lys1* locus by homologous recombination of the truncated *lys1-N* gene on the plasmid and the *lys1-131* point mutation in a *rec12Δ::hygR* deletion strain, and correct integration confirmed by sequencing.

### Spore viability

Parental strains for crosses were grown in YEL plus supplements, cell material was mixed and plated on MEA plus supplements, followed by incubation for three days at 34°C (involving the temperature-sensitive allele *rad50S*). The cross material was then suspended and treated with glusulase [1∶1000(v/v) Helix pomatia juice, Biosepra] solution over night at 30°C. 0.1 ml undiluted or ten-fold diluted spore suspension was plated onto YEA plus supplements. The plates were incubated at 30°C for at least 20 hours. Afterwards the plates were inspected under the microscope of a Singer tetrad dissection apparatus (allows systematic inspection of non-overlapping fields of view) [54]. Two classes of spore fates were distinguished and quantified: one spore/cell and 2–4 cells (no division, no further growth), in comparison to >4 cells (microcolonies). Only the latter contributed to spore viability.

### Rec12-oligonucleotide assay

50 ml cells of OD_595_ 0.8 were harvested from meiotic cultures at the indicated time points (hours after temperature shift to 34°C), washed with water and disrupted with glass beads in 400 µl lysis buffer (50 mM HEPES, 140 mM NaCl, 1 mM EDTA, 1% Triton-X100, 0.1% (w/v) Na deoxycholate, 1 mM Pefabloc SC (Roche), 0.5% (v/v) Pefabloc SC protector solution, Complete Protease Inhibitor tablet (Roche)) using the FastPrepMachine (Bio101) at level 6, three times 30 seconds with at least 2 minutes cooling on ice in between. The crude extract was centrifuged for 10 minutes at 13,000 rpm in a microcentrifuge at 4°C and the supernatant was centrifuged again for 5 minutes. [53]

For each immunoprecipitation (IP) sample, 30 µl Dynabeads Protein G (Invitrogen) were pre-coated in 50 µl 0.1 M citrate-phosphate buffer with 6 µg monoclonal anti-myc antibody (9E10, SantaCruz) or anti-HA (12CA5, Roche), agitated at 22°C. IPs were carried out on a rotating wheel (4°C, 2 hours) by adding the cleared extract directly to the pre-coated beads. Immune complexes were collected with a magnet, washed twice with 1× NEB4 buffer (50 mM K-acetate, 20 mM Tris-acetate, 10 mM Mg-acetate, 1 mM DTT, NewEnglandBiolabs) and incubated in 50 µl 1× terminal transferase labeling buffer (50 mM K-acetate, 20 mM Tris-acetate, 10 mM Mg-acetate, NewEnglandBiolabs) with 2.5 mM CoCl_2_ (NewEnglandBiolabs), 40 U terminal transferase (TdT, NewEnglandBiolabs ) and 8 µCi [alpha ^32^P] cordycepin triphosphate (5000 Ci/mmol, Perkin Elmer) for 2 hours at 37°C with agitation. IP complexes were washed twice with 1× NEB4 buffer. Elution was carried out by boiling in 50 µl 2× Laemmli buffer (100 mM Tris-HCl pH 6.8, 20% glycerol, 1 mM EDTA, 4% SDS, 0.05% bromphenol blue). Rec12-oligonucleotide was separated by SDS-PAGE (10%), fixed, dried, and exposed. Quantification of Rec12-oligonucleotide was done with AIDA 1D- quantification software (raytest). A 4-hour sample from a *pat1-114* meiotic wild-type time-course experiment served as a normalization standard (N). For each time point the amount of Rec12-oligonucleotide was determined by normalizing the amount of radioactivity to the meiotic sample N that was processed in parallel and loaded next to each experiment. The summation of Rec12-oligonuclotide values at each time point in wild type (*pat1-114*) corresponded to 100%. Values of Rec12-oligonucleotide in mutant strains were determined accordingly.

For Western blot analysis, protein was transferred onto a PVDF membrane in transfer buffer (25 mM Tris, 190 mM glycine, 20% methanol), probed with monoclonal anti c-myc-peroxidase antibody (1∶10,000, clone 9E10, Roche), and detected with the ECL Plus Western Blot Detection Kit (Amersham).

### Dot-blot analysis of Rec12myc protein

For detection of Rec12myc abundance during whole time course experiments 10% of immunoprecipitated Rec12 was retained and eluted in 20 µl elution buffer (4% SDS, 100 mM Tris-HCl pH 6.8, 1 mM EDTA) by heating (7 minutes, 97°C). 5 µl were dropped on PVDF membrane and probed with monoclonal anti c-myc-peroxidase antibody (1∶10,000, clone 9E10, Roche), and detected with the ECL Plus Western Blot Detection Kit (Amersham).

### Oligonucleotide length determintation

After TdT treatment, immunocomplexes were eluted in 30 µl Pronase elution buffer (0.5% SDS, 100 mM Tris (pH 7.5), 10 mM CaCl, 10 mM EDTA) by boiling for 5 minutes. Pronase (Roche) was added for deproteinization (final concentration: 0.2 mg/ml, 2 hours at 50°C). After four phenol/chloroform/iso-amylalcohol (25∶24∶1) and one chloroform/iso-amylalcohol extractions, the supernatant containing radiolabeled DNA was then directly mixed with 30 µl formamide loading buffer (80% formamide, 0.1% xylene xyanol, 0.1% bromphenol blue) and boiled for 2 minutes. Extracts from wild type, tagged Rec12 (Rec12myc), and Rec12 active site mutant (Rec12Y98Fmyc), were processed in parallel and the whole sample was separated on a denaturing 20% poly-acrylamid gel. Gels were fixed, dried on Whatman paper prior to exposure.

### Analysis of meiotic DSB formation by PFGE

Genomic DNA embedded in agarose plugs was prepared as described in [53]. The chromosomal DNA in plugs was analyzed by pulsed-field gel electrophoresis (PFGE) as previously described [4].

### Intragenic recombination at *ade7*

Crosses of *h^−^ ade7-50* and *h^+^ ade7-152* were carried out. Appropriate dilutions of spores were plated on selective (MMA) and non-selective media (YEA) and incubated for 5 days at 30°C. Prototroph frequencies were calculated as the number of prototrophs per 10^6^ viable spores (ppm).

## Supporting Information

Figure S1 FACS analysis of *pat1-114* meiotic time courses. Strains were: *rec12-myc* (KLY67), *rec12-myc mek1Δ* (MR442), *rec12-myc hop1Δ* (MR395), *rec12-myc mek1Δ hop1Δ* (MR440), *rec12-myc rad50S* (MR399), *rec12-myc rad50S mek1Δ* (MR450), *rec12-myc rad50S hop1Δ* (MR391), and *rec12-myc rad50S mek1Δ hop1Δ* (MR446). For complete genotypes see Table S1. Nitrogen starved cells were shifted to 34°C to induce synchronous meiosis. Meiotic DNA replication was complete after 2 hours.
(0.77 MB EPS)

Figure S2 Dot-blot analysis showing equal loading of Rec12myc in mutant and wild type strains. Immunoprecipitate samples of Rec12-oligonucleotide experiments shown in Figure 4 were dot-blotted and Rec12myc presence revealed by myc antibody. The strains were: *rec12myc* (KLY67), *rec12myc mek1Δ* (MR442), *rec12myc hop1Δ* (MR395), *rec12myc mek1Δ hop1Δ* (MR440), *rec12myc rad50S* (MR399), *rec12myc rad50S mek1Δ* (MR450), *rec12myc rad50S hop1Δ* (MR391), and *rec12myc rad50S mek1Δ hop1Δ* (MR446).
(1.00 MB EPS)

Table S1 Strain list.
(0.05 MB DOC)
