# Supplementary material for: Ctp1 and the MRN-Complex Are Required for Endonucleolytic Rec12 Removal with Release of a Single Class of Oligonucleotides in Fission Yeast
Source: PLoS Genet. 2009 Nov 13;5(11):e1000722. doi: 10.1371/journal.pgen.1000722 (PMC2768786; doi:10.1371/journal.pgen.1000722)
Supplement: Table S1 — Strain list. (0.05 MB DOC) [file pgen.1000722.s003.doc]

##### Table S1 Strain list

| **Strains** | **Genotype** | **Derivation** |
| --- | --- | --- |
| KLY67 | *h^+^ ade6-M216 pat1-114 rec12-myc13::kanMX* | [53] |
| MR391 | *h^-^ ade6-M210 pat1-114 rec12-myc13::kanMX hop1::kanMX rad50S* | *This study* |
| MR395 | *h^-^ ade6-M210 pat1-114 rec12-myc13::kanMX hop1::kanMX* | *This study* |
| MR399 | *h^-^ ade6-M216 pat1-114 rec12-myc13::kanMX rad50S* | *This study* |
| 22-859 | *h^+^ pat1-114* | *Bern collection* |
| KLY384 | *h^-^ pat1-114 lys1^+^::rec12Y98F-myc13::kanMX rec12::HygR* | *This study* |
| MR440 | *h^+^ ade6-216 pat1-114 hop1::kanMX mek1::kanMX rec12-myc13::kanMX* | *This study* |
| MR442 | *h^-^ ade6-216 pat1-114 mek1::kanMX rec12-myc13::kanMX* | *This study* |
| MR446 | *h^+^ pat1-114 ade6-110 hop1::kanMX mek1::kanMX rec12-myc13::kanMX rad50S* | *This study* |
| MR450 | *h^-^ pat1-114 ade6-M216 mek1::kanMX rec12-myc13::kanMX rad50S* | *This study* |
| 1-26 | *h^-^ ade7-50* | *Bern collection* |
| 51-2011 | *h^+^ ade7-152* | *Bern collection* |
| MR463 | *h^-^ ade7-50 rad50S* | *This study* |
| MR467 | *h^+^ ade7-152 rad50S* | *This study* |
| MR464 | *h^-^ ade7-50 rad50S hop1::kanMX* | *This study* |
| MR468 | *h^+^ ade7-152 rad50S hop1::kanMX* | *This study* |
| MR465 | *h^-^ ade7-50 rad50S mek1::kanMX* | *This study* |
| MR469 | *h^+^ ade7-152 rad50S mek1::kanMX* | *This study* |
| MR466 | *h^-^ ade7-50 rad50S hop1::kanMX mek1::kanMX* | *This study* |
| MR470 | *h^+^ ade7-152 rad50S hop1::kanMX mek1::kanMX* | *This study* |
| MR475^1^ | *h^+^ pat1-114 rec12-myc13::kanMX ctp1::kanMX* | *This study* |
| MR477^1^ | *h^-^ pat1-114 rec12-myc13::kanMX ctp1::kanMX hop1::kanMX* | *This study* |
| MR479^2^ | *h^-^ pat1-114 rec12-myc13::kanMX rad32-D65N* | *This study* |
| MR481 | *h^-^ pat1-114 rec12-myc13::kanMX rad50::kanMX* | *This study* |
| MR482^1^ | *h- pat1-114 rec12-myc13::kanMX hop1::kanMX rad50S ctp1::kanMX* | *This study* |
| MR484^2^ | *h- pat1-114 rec12myc13::kanMX mek1::kanMX rad50S rad32-D65N* | *This study* |
| MR487^2^ | *h- pat1-114 rec12myc13::kanMX mek1::kanMX rad32-D65N* | *This study* |

#####

*^1^ ctp1::kanMX* is a gift of Paul Russell, San Diego.

*^2^ rad32-D65N* is a gift of Edgar Hartsuiker, Sussex.
